# Localization and function of neurosecretory protein GM, a novel small secretory protein, in the chicken hypothalamus

**DOI:** 10.1038/s41598-017-18822-9

**Published:** 2018-01-15

**Authors:** Kenshiro Shikano, Yuki Bessho, Masaki Kato, Eiko Iwakoshi-Ukena, Shusuke Taniuchi, Megumi Furumitsu, Tetsuya Tachibana, George E. Bentley, Lance J. Kriegsfeld, Kazuyoshi Ukena

**Affiliations:** 1 Section of Behavioral Sciences, Graduate School of Integrated Arts and Sciences, Hiroshima University, Higashi, Hiroshima 739-8521 Japan; 2 Department of Integrative Biology and the Helen Wills Neuroscience Institute, University of California at Berkeley, Berkeley, CA 94720-3140 USA; 3 Department of Agrobiological Science, Faculty of Agriculture, Ehime University, Matsuyama, 790-8566 Japan; 4 Department of Psychology and the Helen Wills Neuroscience Institute, University of California at Berkeley, Berkeley, CA 94720-3140 USA

## Abstract

Recently, we discovered a novel cDNA encoding the precursor of a small secretory protein, neurosecretory protein GL (NPGL), in the hypothalamic infundibulum of chickens. NPGL plays an important role in the regulation of growth and feeding. A database search indicated that the *NPGL* gene has a paralogous gene: neurosecretory protein GM (*NPGM*), also in chickens. We identified cDNA encoding the NPGM precursor in chickens. Morphological analysis showed that NPGM-containing cells are specifically localized in the medial mammillary nucleus (MM) and infundibular nucleus (IN) in the hypothalamus. In addition, we found that NPGM and NPGL are co-localized, especially in the MM. The expression levels of *NPGM* mRNA gradually decreased during post-hatch development, in contrast to those of *NPGL* mRNA. Moreover, we investigated the relationship between NPGM and other known factors. NPGM was found to be produced in histaminergic neurons in the MM. NPGM and histidine decarboxylase, a histamine-producing enzyme, displayed similar expression patterns during post-hatch development. Acute intracerebroventricular injection of NPGM decreased food intake, similar to the effect of histamine. To our knowledge, this is the first report of the localization and function of NPGM in the brain of vertebrates. These results will further advance the understanding mechanisms underlying energy homeostasis.

## Introduction

Energy homeostasis is regulated by hormones and neuropeptides released from a variety of sources, such as the gut, liver, adipose tissue, and brain^1^. The hypothalamus integrates peripheral and central signals to monitor the energetic state of the organism. Feeding behavior is a critical component of energy homeostasis and poultry, such as chickens, are often utilized in studies on appetite control. These have improved commercial productivity and have helped us understand common mechanisms underlying energy homeostasis in vertebrates^2^. In avian species, the infundibular nucleus (IN) in the hypothalamic infundibulum is known to possess first-order neurons for feeding regulation^3^. The IN contains orexigenic neuropeptide Y (NPY) and agouti-related protein (AgRP) neurons, which have complementary functions^4,5^. In contrast, the IN also contains anorexigenic pro-opiomelanocortin (POMC) neurons^6^. Second-order neurons for feeding regulation are included in the paraventricular nucleus (PVN), expressing corticotropin-releasing hormone (CRH) and thyrotropin-releasing hormone (TRH)^3,7,8^. In addition, several bioactive factors, including ghrelin and growth hormone-releasing hormone (GHRH), are also involved in ingestive behavior^9^. Avian and mammalian species share many common neuropeptides which maintain energy homeostasis, such as NPY, POMC, CRH, melanin-concentrating hormone (MCH), and ghrelin^10^. However, these peptides do not always play similar roles in birds and mammals^10^. In mammals, MCH, ghrelin and GHRH have orexigenic effects^11–13^. On the other hand, in birds, MCH does not affect food intake, and ghrelin and GHRH have anorexigenic effects^9,10^. The mechanism of appetite control has not been fully understood in vertebrates, partly because of such differences in feeding regulation between birds and mammals^10^.

To further understand the mechanisms regulating energy homeostasis in vertebrates, we sought to identify novel factors involved in energy intake and metabolism. Recently, we identified a novel cDNA encoding the precursor of a small neurosecretory protein in the hypothalamus of chickens, mice, and rats^14–16^. The precursor protein contained a signal peptide sequence, a mature protein sequence, a glycine amidation signal, and a dibasic amino acid cleavage site. Because the predicted C-terminal amino acids of the small protein were Gly-Leu-NH_2_, the small protein was named neurosecretory protein GL (NPGL)^14^. *In situ* hybridization indicated that *NPGL* mRNA was produced in the medial mammillary nucleus (MM) and the IN within the hypothalamic infundibulum of chicken^14^. In addition, *NPGL* mRNA levels were found to have increased during post-hatching development^14^. Chronic subcutaneous and intracerebroventricular (i.c.v.) infusion of NPGL both increased body mass gain in chicks; the latter also increased food intake^14,17^. These findings suggest that NPGL participates in the growth of chicks. Recently, we also found that NPGL stimulates feeding behavior in mice and rats^15,16^.

A genome database search suggested the presence of a paralogous gene, named neurosecretory protein GM (*NPGM*)^14^, and analysis suggested that *NPGM* and *NPGL* are conserved in vertebrates, including chickens, rats, and humans^14^. Therefore, we expected that NPGM would have significant biological functions in vertebrates. However, prior to the present study, it was unclear whether NPGM is even expressed in the brain. To investigate the physiological function(s) of NPGM and the relationship between NPGM and NPGL, we therefore performed cDNA cloning of the NPGM precursor and analyzed its localization and the effects of i.c.v. injection of mature NPGM in chicks.

## Results

### Identification of cDNA encoding NPGM

**Figure 1:**
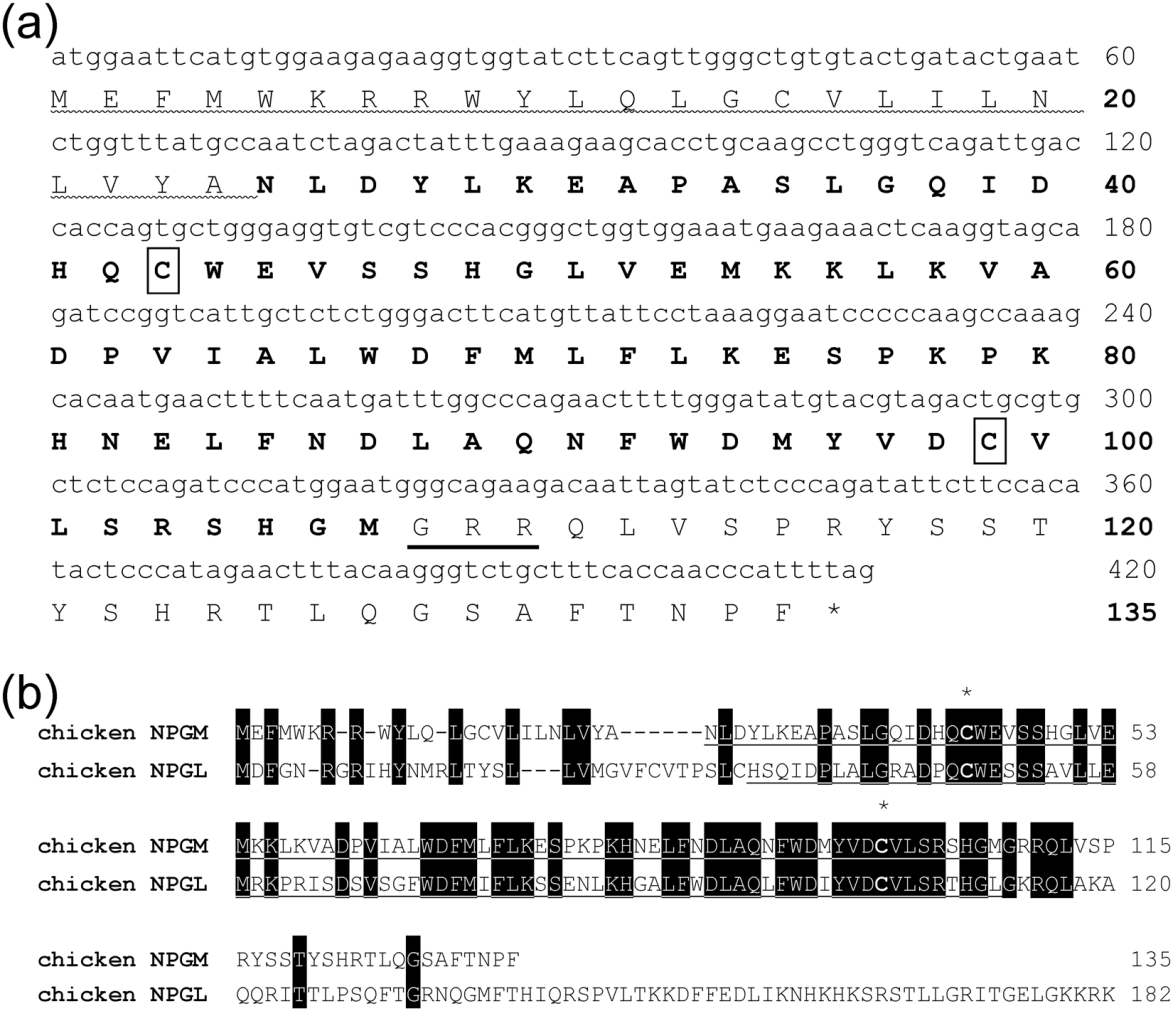
Nucleotide sequence of NPGM precursor and amino acid sequence alignment of NPGM and NPGL precursor proteins. (**a**) The predicted signal peptide is denoted by a wavy line. The sequence of the predicted 83-amino acid residues of the mature small protein is presented in bold face. The Gly (G) C-terminal amidation signal and the Arg (R)-Arg (R) dibasic processing site are underlined. Two Cys (C) residues are boxed. The asterisk indicates the stop codon (TAG). (**b**) Amino acid sequence alignment of NPGM and NPGL precursor proteins deduced from chicken cDNA sequence. Black boxes highlight conserved amino acids. Gaps, indicated by hyphens, were inserted to optimize sequence alignment. Predicted mature small proteins are underlined. The conserved Cys (C) residues are indicated by asterisks.

We performed a BLAST search on a genome database (Ensembl Genome Browser; www.ensembl.org) using the amino acid sequence of the NPGL precursor in chicken. We discovered a paralogous gene for the chicken protein. Using PCR primers based on the database sequences in chicken, we cloned a cDNA spanning the entire coding region of the *NPGM* gene and found it to be identical to that provided in the cDNA database (XM_429770.2). The *NPGM* cDNA is 408 bp long and comprises an open reading frame (ORF) that encodes 135-amino acids. The nucleotide and amino acid sequences of the precursor protein are shown in Fig. 1a. Analysis of the N-terminal sequence of the deduced protein with the SignalP program (www.cbs.dtu.dk/services/SignalP/) revealed the presence of a 24-amino acid signal peptide (Fig. 1a). The predicted 83-amino acid residues of the small protein are flanked at the C-terminus by a dibasic Arg-Arg motif, which consists of a potential proteolytic processing site. The precursor protein possesses a Gly residue at its C-terminal end that contributes to amidation (Fig. 1a). Therefore, the predicted C-terminal amino acid sequences of the small protein are Gly-Met-NH_2_. As a result, this small protein was termed neurosecretory protein GM (NPGM). The mature NPGM contains two Cys residues, suggesting an intramolecular disulfide bond formation (Fig. 1a). Mature NPGM in chicken shares a relatively high amino acid sequence identity (54%) with chicken NPGL, but it does not share any amino acid sequence identity with other known neuropeptides (Fig. 1b).

### Distribution of the NPGM precursor mRNA and mature protein

**Figure 2:**
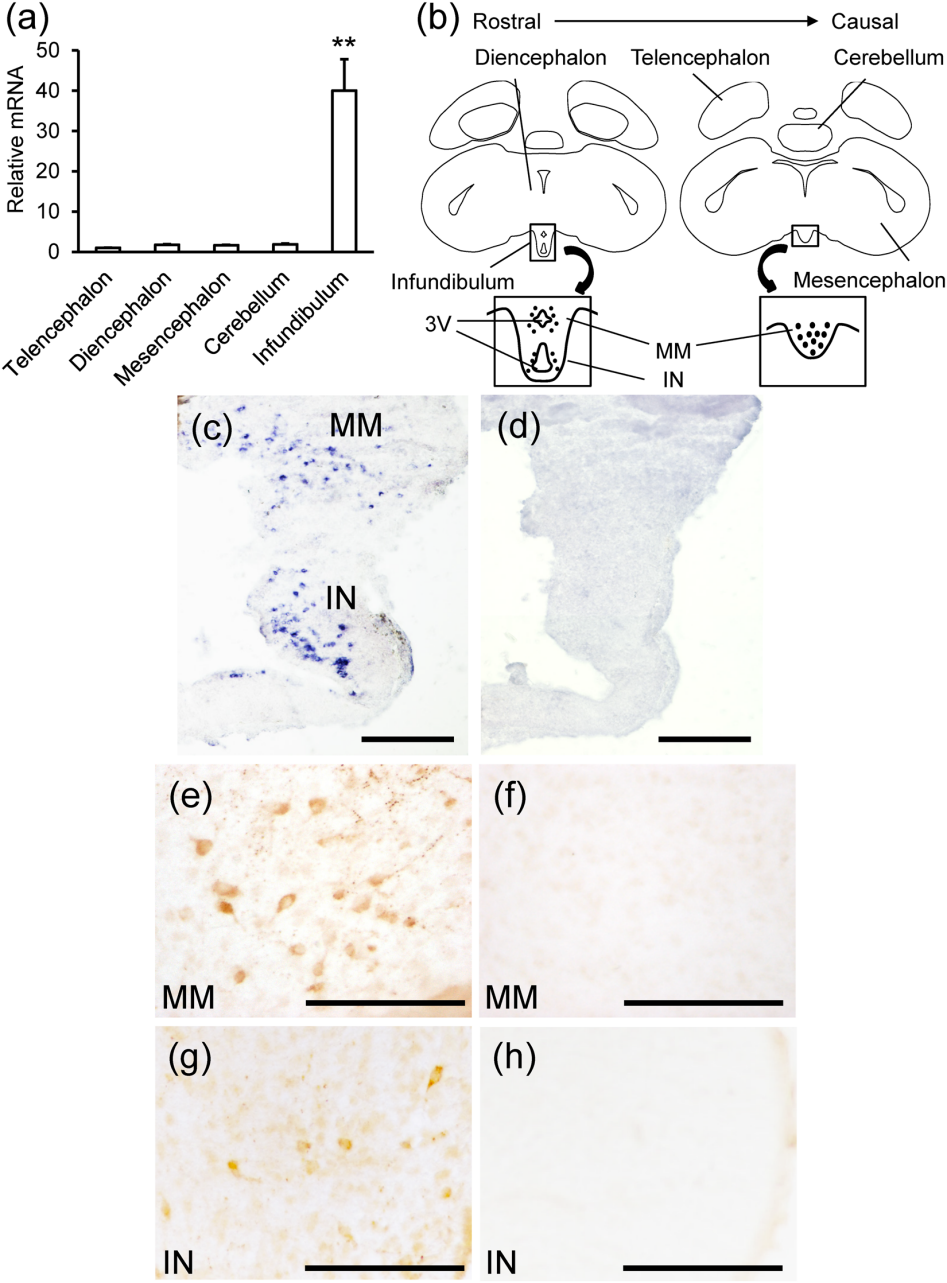
Localization of NPGM mRNA and the mature protein in the hypothalamic infundibulum. (**a**) Real-time PCR analysis of the NPGM precursor mRNA concentrations in different brain regions. Each value represents the mean ± SEM (n = 5). The asterisk indicates a statistically significant difference (One-way ANOVA with Tukey’s test as a post-hoc test: ^**^P < 0.01). (**b**–**d**) Schematic representation and photomicrographs illustrating the distribution of cells expressing NPGM precursor mRNA in the hypothalamic infundibulum. (**b**) The location of the hypothalamic infundibulum is shown in the coronal brain illustration. Cells expressing the NPGM precursor mRNA and mature NPGM in the medial mammillary nucleus (MM) and the infundibular nucleus (IN) within the hypothalamic infundibulum are represented by dots in the illustration. (**c**) Cells expressing the NPGM precursor mRNA in the MM and the IN were obtained from 1-day-old chicks. (**d**) No signals were detected by the sense probe. (**e**–**h**) Photomicrographs of NPGM-immunoreactive cells and fibers. NPGM-immunoreactive perikarya in the MM (**e**) and the IN (**g**). No signals were detected by the antibody adsorption test (**f**,**h**). Scale bar = 100 µm.

The expression levels of the NPGM precursor mRNA were examined in 1-day-old chicks by real-time PCR using samples from different brain regions, such as the telencephalon, diencephalon, mesencephalon, cerebellum, and hypothalamic infundibulum. NPGM precursor mRNA was expressed only in the hypothalamic infundibulum; its expression in other regions of the brain was not detectable (Fig. 2a). *In situ* hybridization data indicated that *NPGM* mRNA-containing cells were localized in the MM and the IN within the hypothalamic infundibulum in 1-day-old chicks (Fig. 2b and c). Additionally, *NPGM* mRNA was expressed predominantly in the posterior region of the MM (Fig. 2b). No signal was detected with the sense probe (Fig. 2d). Immunohistochemical analysis indicated presence of NPGM-immunoreactive cells and fibers in the MM and the IN; this result was replicated in terms of mRNA expression, assessed via *in situ* hybridization (Fig. 2b,e and g). The immunoreactive signals were blocked by the neutralization of the primary antibody pre-incubated with synthetic mature NPGM (Fig. 2f and h).

### Expression of *NPGM* and *NPGL* mRNA during post-hatch development

**Figure 3:**
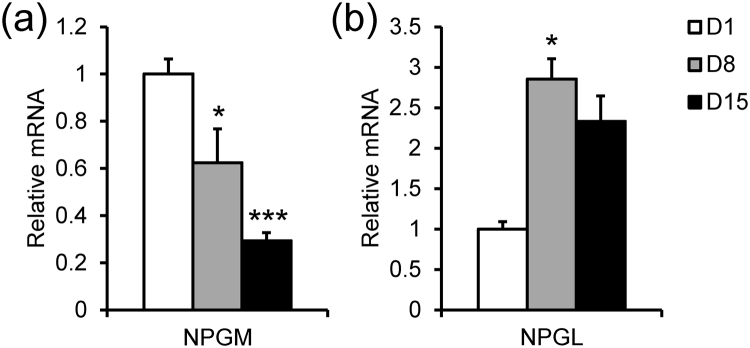
Expression level of *NPGM* and *NPGL* mRNA during post-hatch development using real-time RT-PCR. Real-time RT-PCR analysis of NPGM (**a**) and NPGL (**b**) precursor mRNA in the hypothalamic infundibulum in 1-, 8-, or 15-day-old chicks. Data are expressed as mean ± SEM (n = 5–6). The asterisk indicates a statistically significant difference versus 1-day-old (One-way ANOVA with Tukey’s test as a post-hoc test: *P < 0.05, ***P < 0.005 vs. D1).

**Figure 4:**
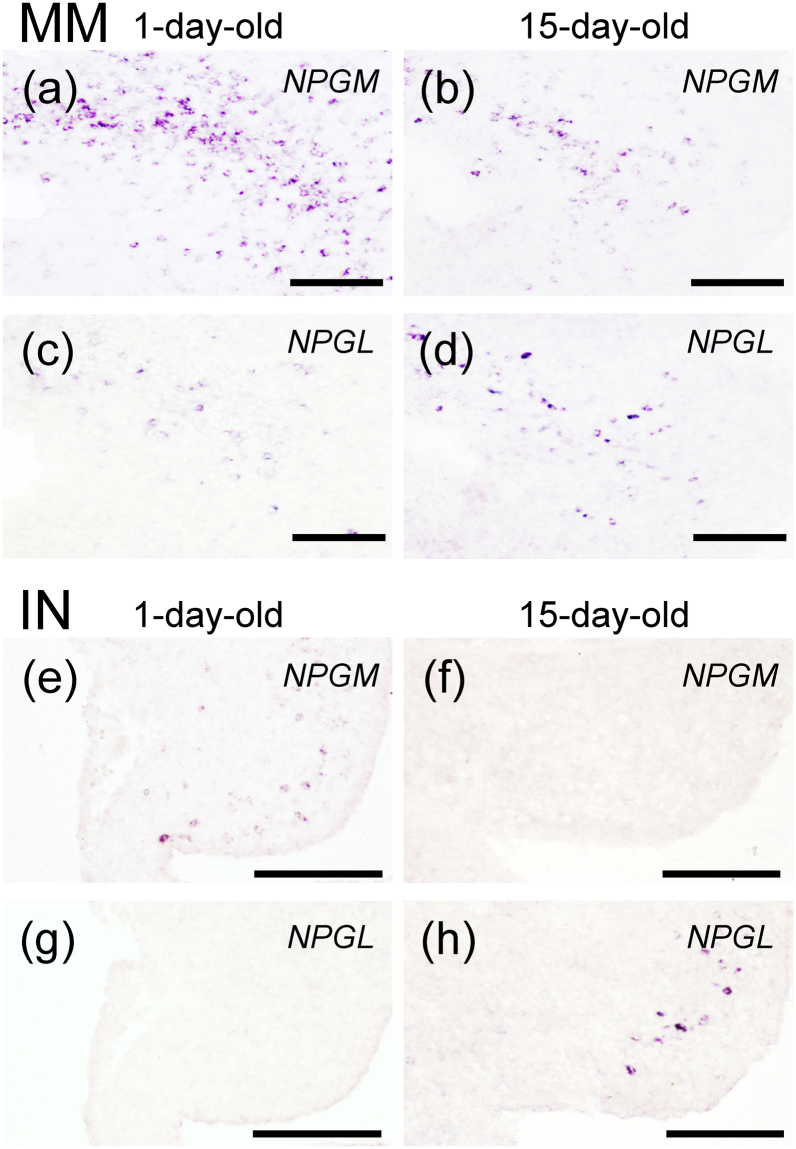
Localization of *NPGM* and *NPGL* mRNA-expressing cells in 1- and 15-day-old chicks using *in situ* hybridization. (**a**–**h**) Photomicrographs of cells expressing the NPGM (**a**,**b**,**e**,**f**) and NPGL (**c**,**d**,**g**,**h**) precursor mRNA in the medial mammillary nucleus (MM) (**a**–**d**) and the infundibular nucleus (IN) (**e**–**h**) were obtained after performing *in situ* hybridization with coronal brain sections from 1-day-old (**a**,**c**,**e**,**g**) and 15-day-old (**b**,**d**,**f**,**h**) chicks. Scale bar = 100 µm.

To investigate the common and/or different features between NPGM and NPGL, we analyzed the NPGM and NPGL precursor mRNA levels in the hypothalamic infundibulum including the MM and the IN, during post-hatch development by real-time PCR using 1-, 8-, and 15-day-old chicks. *NPGM* mRNA expression levels gradually decreased during development (Fig. 3a). In contrast, *NPGL* mRNA expression levels were elevated in 8-day-old chicks compared to 1-day-old (Fig. 3b). *In situ* hybridization also showed that *NPGM* mRNA was highly expressed in the MM and the IN after hatching, and that this expression decreased from day 1 to day 15 (Fig. 4a,b,e and f). In contrast, *NPGL* mRNA expression in the MM and the IN increased from day 1 to day 15 (Fig. 4c,d,g and h). Although *NPGM* and *NPGL* mRNA showed opposite expression patterns during post-hatch development, both mRNAs were expressed in similar locations in the MM at the same developmental stage.

### Co-localization of NPGM and NPGL

**Figure 5:**
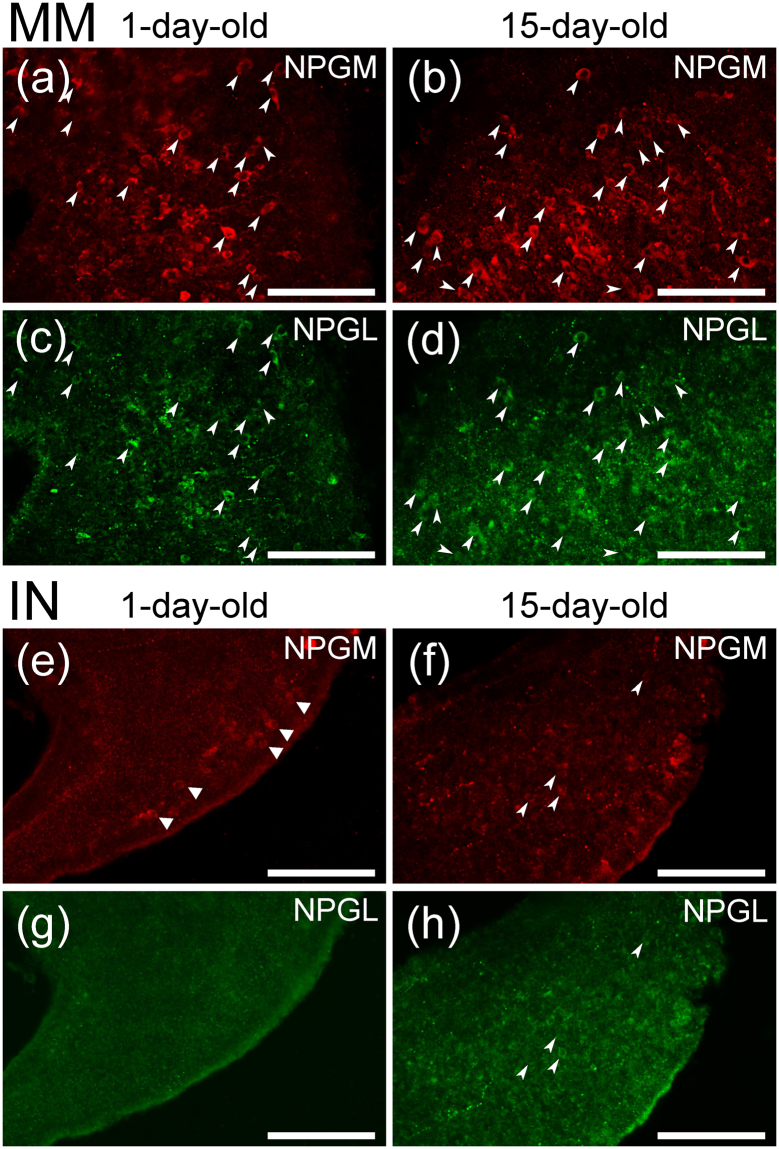
Localization of NPGM and NPGL neurons in 1- and 15-day-old chicks using immunohistochemistry. (**a–h**) Photomicrographs of NPGM- (**a**,**b**,**e**,**f**) and NPGL- (**c**,**d**,**g**,**h**) immunoreactive cell bodies and fibers in the medial mammillary nucleus (MM) (**a**–**d**) and the infundibular nucleus (IN) (**e**–**h**) were obtained after performing immunohistochemistry with coronal brain sections from 1-day-old (**a**,**c**,**e**,**g**) and 15-day-old (**b**,**d**,**f**,**h**) chicks. Arrow-heads indicate NPGM- and NPGL-coexpressing neurons. Triangles indicate only NPGM-producing neurons. Scale bar = 50 µm.

By immunohistochemical analysis with specific antibodies (Supplementary Fig. S1), we further investigated whether NPGM and NPGL were produced in the same neurons in the hypothalamic infundibulum. Approximately 30–40% NPGM-immunoreactive cells were co-localized with NPGL-immunoreactive cells in the MM in 1-day-old chicks (Fig. 5a and c and Supplementary Fig. S2a). Almost all NPGM-immunoreactive cells were co-localized with NPGL-immunoreactive cells in the MM in 15-day-old chicks (Fig. 5b and d and Supplementary Fig. S2b). Although some NPGM-immunoreactive cell bodies were detected in the IN in 1-day-old chicks, there were no NPGL-immunoreactive cell bodies in the IN (Fig. 5e and g). By 15 days of age, a few NPGM-immunoreactive cells had become co-localized with NPGL-immunoreactive cells in the IN (Fig. 5f and h and Supplementary Fig. S2c). In addition, many dense NPGL-immunoreactive fibers were distributed around immunoreactive cells in the MM and the IN in 15-days-old chicks (Fig. 5d and h and Supplementary Fig. S2b and c). Taken together, NPGM-producing cells also produced NPGL in the MM in 1- or 15-day-old chicks, and the ratio of co-localization between NPGM and NPGL increased during post-hatch development. In contrast, in the IN, almost no cells produce both NPGM and NPGL in either 1- or 15-day-old chicks.

### Relationship between NPGM and histamine

**Figure 6:**
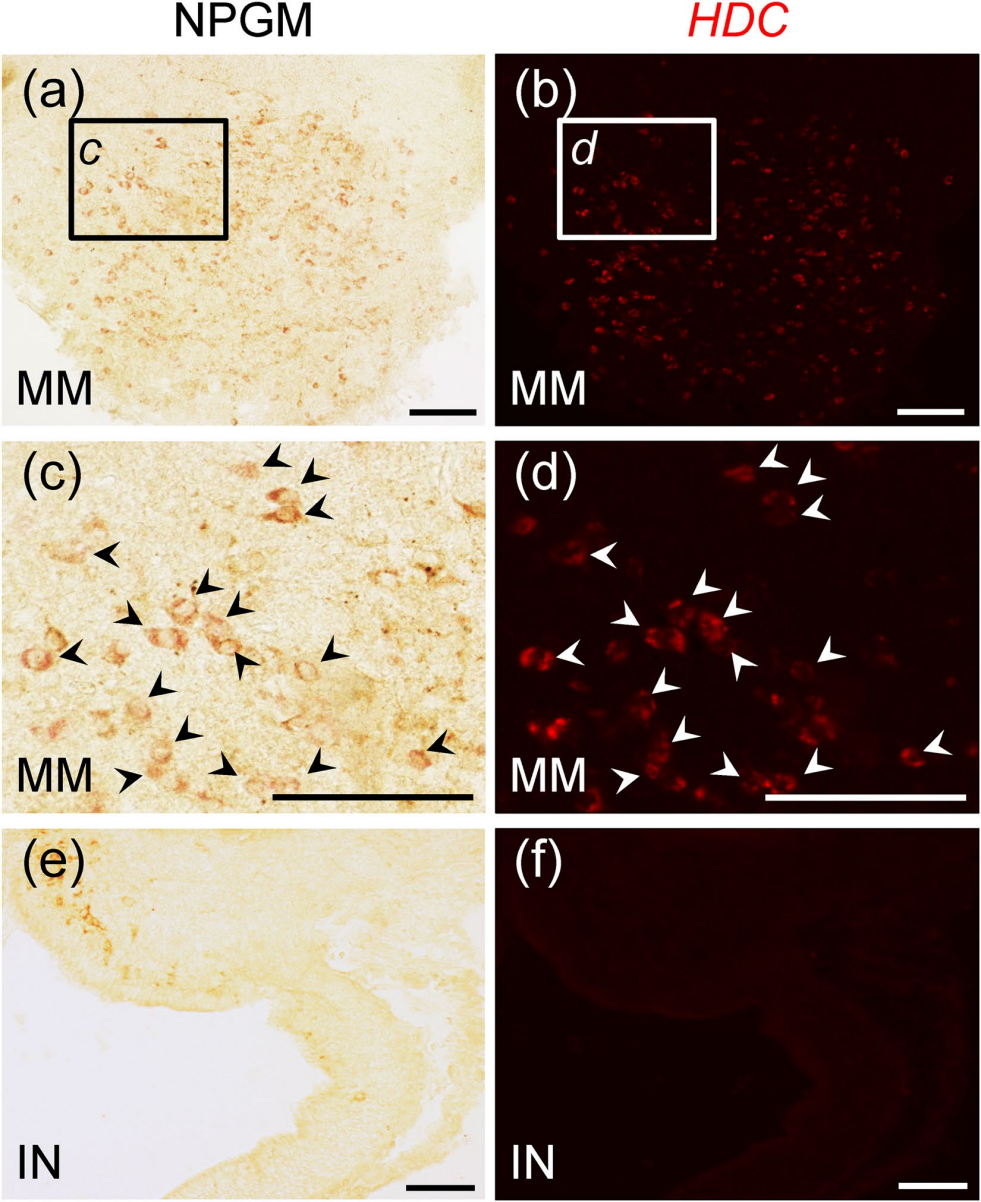
Co-localization of NPGM and histamine neurons. (**a**,**c**,**e**) NPGM-immunoreactive cells are localized in the medial mammillary nucleus (MM) (**a**,**c**) and the infundibular nucleus (IN) (**e**). (**b**,**d**,**f**) HDC precursor mRNA expressing cells are localized in the MM (**b**,**d**) and the IN (**f**). Arrow-heads indicate NPGM- and HDC-coexpressing neurons. Scale bar = 100 µm.

We revealed co-localization of NPGM and NPGL, especially in the MM. Furthermore, we investigated the possibility of co-localization with several other bioactive factors expressed in the hypothalamus. From our preliminary and previous experimental data, we considered it likely that the localization of NPGM-containing cells was close to that of the histaminergic neurons in the posterior region of the MM^18^. It was also clear that NPGM neurons are distinct from neurotensin, gonadotropin-inhibiting hormone (GnIH), and 26RFa (QRFP) neurons, as these are localized in other regions of hypothalamus^19–21^. Therefore, we investigated the relationship between NPGM and histamine by double-labeling using immunohistochemistry and *in situ* hybridization. We found that NPGM-immunoreactive cells were localized in the MM and the IN (Fig. 6a,c and e), but that the precursor mRNA for histidine decarboxylase (HDC), a histamine-producing enzyme, was only expressed in the MM (Fig. 6b,d and f). All NPGM-immunoreactive cells in the posterior MM were identical to HDC precursor mRNA-containing cells (Fig. 6c and d). Subsequently, we measured the *HDC* mRNA expression level during post-hatch development. *HDC* mRNA expression gradually decreased with post-hatch development (Supplementary Fig. S3). Therefore, the expression of *HDC* mRNA showed a similar developmental pattern to that of *NPGM* mRNA (Fig. 3a).

### Effect of i.c.v. injection of NPGM

**Figure 7:**
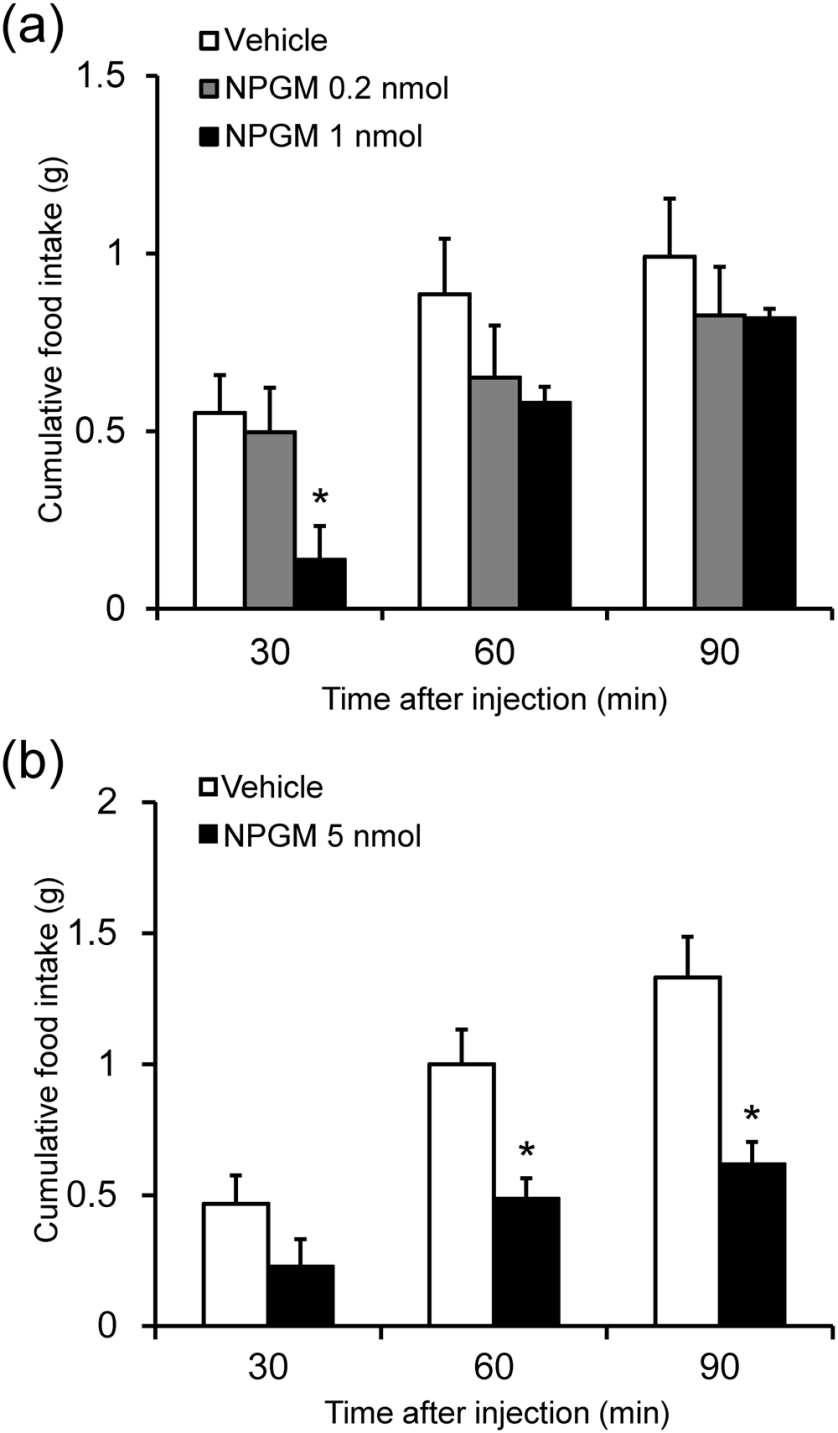
Effect of intracerebroventricular (i.c.v.) injection of NPGM on food intake in 5- or 6-day-old chicks. (**a**) Cumulative food intake after i.c.v. injection of vehicle or NPGM (0.2 and 1 nmol) in 6-day-old chicks. (**b**) Cumulative food intake after i.c.v. injection of vehicle or NPGM (5 nmol) in 5-day-old chicks. Data are expressed as mean ± SEM (n = 8). The asterisk indicates a statistically significant difference versus vehicle (One-way ANOVA with Tukey’s test as a post-hoc test or Student’s *t*-test: *P < 0.05 vs. Vehicle).

We investigated the effect of NPGM on the feeding behavior of 5- or 6-day-old chicks. I.c.v. injection of 1 nmol NPGM immediately decreased food intake for 30 min after injection (Fig. 7a). Additionally, i.c.v. injection of 5 nmol NPGM remarkably decreased food intake up to 90 min after injection (Fig. 7b). These results show that NPGM decreases feeding behavior in a dose-dependent manner (0.2 nmol NPGM had no significant effect).

## Discussion

After the identification of a cDNA encoding NPGL in the hypothalamus of chicken, we found a paralogous gene, *NPGM*, in the same animal^14^. Therefore, in the present study we attempted to characterize NPGM in the chicken hypothalamus. The sequences of NPGM and NPGL were found to be highly conserved, and these small proteins have no homology with previously known peptides. The NPGM precursor mRNA and protein were expressed in the MM and the IN in the hypothalamic infundibulum. Although immunohistochemical analysis indicates the existence of endogenous NPGM protein, we have yet to confirm the presence of mature NPGM using another analysis, such as mass spectrometry. The expression of *NPGM* mRNA gradually decreased during post-hatching development, in contrast to the expression of *NPGL* mRNA. Subsequently, we investigated the potential for co-localization of NPGM and NPGL. Co-localization of NPGM and NPGL was observed particularly in the MM, with almost none in the IN. The different developmental patterns and localization of NPGM and NPGL in the IN suggest that they may have different roles. In fact, we previously found that NPGL induces orexigenic activity in chicks^17^, whereas NPGM was found to exhibit an anorexigenic effect in this study. In mammals, POMC is a precursor protein of α-melanocyte stimulating hormone (α-MSH) and β-endorphin, which have anorexigenic and orexigenic effect, respectively. POMC neurons are generally known as anorexigenic neurons, while it has been reported that POMC neurons promote cannabinoid induced feeding via upregulation of β-endorphin^22^. In this way, anorexigenic neurons could also produce an orexigenic factor. Although it remains to be elucidated which NPGM neurons in the MM or the IN contribute to control feeding behavior, it is quite possible that anorexigenic and orexigenic activities could be generated from the same NPGM/NPGL neurons. Future studies are necessary to reveal which particular NPGM neurons in the MM or the IN contribute to regulate feeding behavior. Furthermore, because the expression of NPGM alters during development, and because we used chicks of a single age (5- or 6-day-old) for i.c.v. injections in the present study, it would be interesting to investigate the effect of NPGM on food intake in chicks of different ages (1-day-old to adults).

NPGM-immunoreactive fibers were mainly observed in the hypothalamus, including the MM and the IN. Within the MM and the IN, some neuropeptides including NPY and α-MSH, derived from POMC, participate in a variety of physiological functions^3–6^. To investigate the correlation between NPGM and known factors, we analyzed the localization of several bioactive substances, such as neurotensin, GnIH, 26RFa (QRFP), and histamine through our previous and preliminary experiments^18–21^. We found that that NPGM-expressing cells are localized close to histaminergic neurons. Recently, we characterized HDC, which is a rate-limiting enzyme for histamine synthesis in chicken^18^. As a result, we found that neuronal histamine is produced in the MM and i.c.v. injection of histamine suppresses feeding behavior^18^. In the present study, morphological analysis showed that NPGM-containing neurons might also produce histamine in the MM. These results suggest that NPGM, histamine, and NPGL are produced in the same cells in the MM in chicks. In mammals, it has been demonstrated that neuronal histamine contributes to various functions, namely, feeding, energy homeostasis, sleep/wakefulness, motor behavior, and cognition^23–25^. For instance, it has been suggested that histaminergic neurons are activated by fasting^26^. Furthermore, histamine neurons are also activated by NPY and may terminate feeding elicited by NPY in mice^27^. This result suggests that histaminergic neurons in the MM maintain basal levels of food intake in birds. Consequently, NPGM produced in histaminergic neurons may act to maintain feeding behavior.

The IN in the avian hypothalamic infundibulum is known to be related to regulation of feeding and growth via NPY/AgRP, POMC, GHRH neurons, and a few other candidates^4–6,28^. Therefore, NPGM in the IN may be related to these functions. In this study, we showed that NPGM suppressed feeding, whereas NPGL induced feeding and body growth in chicks^14,17^. There was almost no co-localization of NPGM and NPGL in the IN. In addition, the expression of *NPGM* and *NPGL* mRNAs during post-hatch development showed opposite patterns. Thus, the function, localization and developmental expression pattern of NPGM in the IN is different from that of NPGL. It is well known that food intake in chicks increases in association with body growth. It is possible that a decrease in anorexigenic NPGM expression, and an increase in orexigenic NPGL expression combine to influence post-hatch development. Future studies on the developmental functions of NPGM and NPGL in individual nuclei, the IN, or the MM, will elucidate complex feeding mechanisms in chicks.

In summary, we have characterized a cDNA encoding a novel secretory protein, NPGM, in the chicken hypothalamic infundibulum. We have revealed the distribution and co-localization of NPGM with NPGL and histamine in the MM. NPGM and histamine display a similar expression pattern during post-hatch development and both have anorexigenic action. Although various factors regulating energy homeostasis have been discovered, including NPY, AgRP, α-MSH, and CRH in birds, the mechanisms of energy homeostasis cannot be explained by these factors alone. We expect that a further investigation of NPGM and NPGL will lead to a greater understanding of the mechanisms regulating energy homeostasis.

## Methods

### Animals

Experimental protocols were approved by the Institutional Animal Care and Use Committee of Hiroshima University (permit number: C13-13) and were in accordance with the Guide for the Care and Use of Laboratory Animals prepared by Hiroshima University.

Male layer chicks (*Gallus domesticus*, 1-day-old) were purchased from a commercial company (Nihon Layer, Gifu, Japan) and housed in a windowless room, maintained at 28 °C on a 20-hour light (4:00–24:00): 4-hour dark (0:00–4:00) cycle. Chicks were provided food and tap water *ad libitum*.

### RNA and cDNA preparation

Chicks were sacrificed by decapitation. Telencephalon, diencephalon, mesencephalon, cerebellum, and hypothalamic infundibulum were dissected out and snap-frozen in liquid nitrogen for further RNA processing. Total RNA from chicken brain tissues was extracted using TRIzol reagent or an RNAqueous-Micro kit (Life Technologies, Carlsbad, CA) in accordance with the manufacturer’s instructions. First-strand cDNA was synthesized from total RNA using a ReverTra Ace kit (TOYOBO, Osaka, Japan). These experiments were performed according to the manufacturer’s instructions.

### Cloning the full-length cDNA of chicken NPGM from the chick hypothalamus

The method of cloning full-length cDNA was similar to that described in a previous report^21^. We first found the sequence of *NPGM* as the paralogous gene of *NPGL* by a BLAST search. We performed molecular cloning of the full-length cDNA. Real-time PCR showed that *NPGM* mRNA was highly expressed in the hypothalamic infundibulum, as described in Fig. 2a. Therefore, cDNA synthesized from the mRNA extracted from the hypothalamic infundibulum was used as a source for cloning. Primers for cloning full-length cDNA were designed from the predicted nucleotide sequences in the NPGM as follows; sense primer: 5′-ATGGAATTCATGTGGAAGAG-3′ (nucleotide no. 1 to 20 from the ATG initiation codon) and anti-sense primer: 5′-AGCATCTACAGTAAATGCTG-3′ (nucleotide no. 474 to 493). Nucleotide sequences were determined using an ABI PRISM BigDye Terminator Ready Reaction Cycle Sequencing kit (Applied Biosystems, Foster City, CA), an ABI PRISM 310 Genetic Analyzer (Applied Biosystems), and DNASIS Pro software (Hitachi Software Engineering, Kanagawa, Japan).

### Quantitative RT-PCR

PCR amplifications were conducted with the THUNDERBIRD SYBR qPCR Mix (TOYOBO) using the following procedure: 95 °C for 20 s, followed by 40 cycles at 95 °C for 3 s and at 60 °C for 30 s using Step One Real-time Thermal Cycler (Applied Biosystems). The primer sequences are listed in Supplementary Table 1-1. Relative quantification for each expression was determined by the 2^−ΔΔCt^ method using β-actin (*ACTB*) as an internal control^29^.

### *In situ* hybridization for the detection of NPGM and NPGL

The chicks (1-day or 15-day-old) were killed by decapitation. Their brains were fixed in 4% paraformaldehyde (PFA) solution overnight at 4 °C and then placed in a refrigerated solution (30% sucrose in 10 mM phosphate-buffer, pH 7.4) until they sank. The tissues were embedded in Tissue-Tek OCT Compound (Sakura Finetek, Tokyo, Japan) and sectioned coronally at a thickness of 14 µm with a cryostat at −20 °C. The sections were then mounted on slides. Digoxigenin (DIG)-labeled antisense and sense RNA probes were produced from a section of the small protein precursor cDNA sequence using a DIG RNA Labeling kit with SP6 and T7 polymerases (Roche Diagnostics, Basal, Switzerland). RNA probe labeling was performed according to the manufacturer’s instructions. The primer sequences are listed in Supplementary Table 1-2. Single staining with nitro blue tetrazolium/5-bromo-4-chloro-3-indolyl phosphate (NBT/BCIP) was performed as previously described^21^. Hybridizations were performed overnight at 60 °C or 55 °C for NPGM or NPGL, respectively. The sections were incubated with blocking solution [1% bovine serum albumin, BSA; 1% normal goat serum; 0.3% Triton-X100 in phosphate buffered saline (PBS)], followed by incubation with alkaline phosphatase-labeled anti-DIG Fab fragments (1: 1000 dilution, 1 093 274; Roche) in blocking solution for 1 h. Signals were detected after immersion of the sections overnight in the NBT/BCIP solution. The DIG labeled sense probe was used as a control to verify specificity.

### Production of chicken NPGM and antibodies

The procedure for the production of recombinant NPGM was similar to that described previously^30^. We prepared NPGM combined with the elongated C-terminal Gly (NPGM-Gly) as a recombinant protein using the *Escherichia coli* (*E. coli*) expression system. We transformed *E. coli* using the pCold-TF expression vector (TaKaRa Bio, Shiga, Japan) containing the *NPGM* sequence. The recombinant small protein was purified on a Ni^2+^ column (Qiagen, Venlo, Netherlands), and His_6_-TF-tag was removed with Factor Xa (New England Biolabs, Ipswich, MA). The disulfide bond bridge was formed by potassium ferricyanide. The small protein was purified by reverse phase high-performance liquid chromatography (RP-HPLC) using C8 column (10 × 150 mm; YMC, Kyoto, Japan) at a flow rate of 0.5 mL/min for 40 min with a linear gradient of 40–60% acetonitrile containing 0.1% trifluoroacetic acid. The solvent was evaporated and lyophilized. The C-terminus of recombinant NPGM-Gly was amidated using an amidating enzyme, and the mature form was finally purified by RP-HPLC.

Rabbit antisera against recombinant NPGM were produced following our published method^21^. NPGM solution was mixed with Freund’s complete adjuvant and injected into four rabbits. After a booster injection, blood was collected from the rabbits. Optimal serum with the highest titer was selected via a dot-blot analysis and the antibody specificity of NPGM or NPGL was confirmed via enzyme-linked immunosorbent assay (ELISA). The NPGL antibody was prepared in guinea pig as described previously^17^. Competitive ELISA was performed following our published method^21^. In brief, different concentrations of synthetic NPGM or NPGL (0.01–1000 pmol) were added with rabbit anti-NPGM antibody (1: 1000 dilution) or guinea pig anti-NPGL antibody (1: 1000 dilution) to each antigen-coated well of a 96-well microplate (96-well microplates PS F-bottom; Greiner Bio-One, Frickenhausen, Germany), and incubated for 1 h at 37 °C. After reaction with alkaline phosphatase-labeled goat anti-rabbit IgG (1: 1000 dilution, AP-1000; Vector Laboratories, Burlingame, CA) or anti-guinea pig IgG (1: 1000 dilution, ab7140; abcam, Cambridge, MA), immunoreactive products were obtained in a substrate solution of p-nitrophenylphosphate, and the absorbance was measured at 405 nm on a microtiter plate reader (Multiskan GO; Thermo Fisher Scientific, Kanagawa, Japan).

### Immunohistochemistry (IHC) for the detection of NPGM and NPGL

The chicks (1-day or 15-day-old) were sacrificed by decapitation. The brain fixation method was performed as described above; the brain was sectioned into 14-µm slices. The sections were then mounted on slides and incubated with 0.3% hydrogen peroxide (H_2_O_2_) in absolute methanol for 30 min, for the immunoenzyme technique. After blocking with blocking solution (1% normal goat serum and 1% BSA in PBS containing 0.3% Triton X-100) for 1 h, the sections were incubated with rabbit anti-NPGM antibody (1: 1000 dilution) overnight at 4 °C. The primary immunoreaction was executed by incubation with biotinylated goat anti-rabbit IgG (Vector Laboratories, Burlingame, CA) as a secondary antibody. Immunoreactivity was detected with an ABC kit (VECTASTATIN Elite Kit; Vector Laboratories), followed by diaminobenzidine (DAB) reaction. Preadsorption was performed as a control using the antigen (10 µg/mL). The antigen and primary antibody were preadsorbed overnight prior to the running of immunohistochemistry in the same way as for the non-control slides.

In the case of double immunohistochemical analysis of NPGM and NPGL, rabbit anti-NPGM antibody (1: 1000 dilution) and guinea pig anti-NPGL antibody (1:1000 dilution), as primary antibodies, Cy3-conjugated donkey anti-rabbit IgG (1: 1000 dilution, 711-165-152; Jackson ImmunoResearch, West Grove, PA) for the detection of NPGM, and Alexa Fluor 488-conjugated donkey anti-guinea pig IgG (H + L) (1: 1000 dilution, 706-545-148; Jackson ImmunoResearch) for the detection for NPGL, as secondary antibodies, were used.

### Double staining of HDC and NPGM using *in situ* hybridization and immunohistochemistry

We detected HDC precursor mRNA and mature NPGM using *in situ* hybridization and immunohistochemistry, respectively. Brain sections were hybridized using DIG-labeled probe overnight at 50 °C for the detection of HDC, as described previously^18^. The hybridization protocol of *in situ* hybridization was the same until the hybridization, as described above. The primer sequences are listed in Supplementary Table 1-2. After hybridization, we carried out immunohistochemistry of NPGM with the same method, as described above. After the DAB reaction, DIG was detected by the HNPP Fluorescent Detection Set (Roche).

### Intracerebroventricular (i.c.v.) injection

Five or six-day-old chicks were injected i.c.v. with NPGM according to a previously reported method^31^. The head of the chick was inserted into an acrylic box with a hole in the top plate. The injection coordinates targeting the left lateral ventricle were 3 mm anterior from the parietal bone, 1 mm lateral from the sagittal suture, and 3 mm ventral from the surface of the skull. NPGM (0.2, 1.0, and 5.0 nmol/10 µl) was dissolved in 30% propylene glycol at pH 8.0. The NPGM solution was injected through the hole using a micro-syringe at a volume of 10 µL.

### Statistical analysis

Data were analyzed using Student’s *t*-test for two groups. In addition, data from three or five groups were statistically analyzed using one-way analysis of variance with Tukey’s test as a post-hoc test, as appropriate. The statistical significance level was set at P < 0.05. All results are presented as the mean ± standard error of the mean (SEM).

## Electronic supplementary material

Supplementary Info
