# Author Correction: Localization and function of neurosecretory protein GM, a novel small secretory protein, in the chicken hypothalamus

**DOI:** 10.1038/s41598-018-24103-w

**Published:** 2018-04-13

**Authors:** Kenshiro Shikano, Yuki Bessho, Masaki Kato, Eiko Iwakoshi-Ukena, Shusuke Taniuchi, Megumi Furumitsu, Tetsuya Tachibana, George E. Bentley, Lance J. Kriegsfeld, Kazuyoshi Ukena

**Affiliations:** 1 Section of Behavioral Sciences, Graduate School of Integrated Arts and Sciences, Hiroshima University, Higashi, Hiroshima, 739-8521 Japan; 2 Department of Integrative Biology and the Helen Wills Neuroscience Institute, University of California at Berkeley, Berkeley, CA 94720-3140 USA; 3 Department of Agrobiological Science, Faculty of Agriculture, Ehime University, Matsuyama, 790-8566 Japan; 4 Department of Psychology and the Helen Wills Neuroscience Institute, University of California at Berkeley, Berkeley, CA 94720-3140 USA

Correction to: *Scientific Reports* 10.1038/s41598-017-18822-9, published online 15 January 2018

This Article contains errors in Reference 17 which was incorrectly given as:

Shikano, K. *et al*. Effects of chronic intracerebroventricular infusion of neurosecretory protein GL on body mass and food and water intake in chicks. *Gen. Comp. Endocrinol* **256**, 37–42 (2017).

The correct reference is listed below as ref.^1^.
