# Supplementary material for: Localization and function of neurosecretory protein GM, a novel small secretory protein, in the chicken hypothalamus
Source: Sci Rep. 2018 Jan 15;8:704. doi: 10.1038/s41598-017-18822-9 (PMC5768754; doi:10.1038/s41598-017-18822-9)
Supplement: Supplementary file 1 — Supplementary Info [file 41598_2017_18822_MOESM1_ESM.pdf]

Supplementary Information

Title: Localization and function of neurosecretory protein GM, a novel small secretory protein, in the chicken hypothalamus

Authors: Kenshiro Shikano[1], Yuki Bessho[1], Masaki Kato[1], Eiko Iwakoshi-Ukena[1,2], Shusuke Taniuchi[1], Megumi Furumitsu[1], Tetsuya Tachibana[3], George E. Bentley[2], Lance J. Kriegsfeld[4] & Kazuyoshi Ukena[1,4*]

Affiliations: [1] Section of Behavioral Sciences, Graduate School of Integrated Arts and Sciences, Hiroshima University, Higashi-Hiroshima 739-8521, Japan.

[2] Department of Integrative Biology and the Helen Wills Neuroscience Institute, University of California at Berkeley, Berkeley, CA 94720-3140, USA.

[3] Department of Agrobiological Science, Faculty of Agriculture, Ehime University, Matsuyama 790-8566, Japan.

[4] Department of Psychology and the Helen Wills Neuroscience Institute, University of California at Berkeley, Berkeley, CA 94720-3140, USA.

*To whom correspondence should be addressed.

E-mail: ukena@hiroshima-u.ac.jp

Supplemental Figure Legends

Supplemental Figure 1. Specificity of anti-NPGM and anti-NPGL antibody. (**a**, **b**) Competitive ELISA with anti-NPGM (**a**) and anti-NPGL (**b**) antibody (Ab) against synthetic NPGM and NPGL, respectively.

Supplemental Figure 2. Co-localization of NPGM and NPGL neurons. (**a**−**c**) Merged images of NPGM and NPGL neurons in 1-day-old (**a**) and 15-day-old (**b**, **c**) chicks in the medial mammillary nucleus (MM) (**a**, **b**) and the infundibular nucleus (IN) (**c**). Arrow-heads indicate NPGM- and NPGL-coexpressing neurons. Scale bar = 25 μm.

Supplemental Figure 3. Expression level of *HDC* mRNA during post-hatch development. The expression level of *HDC* mRNA in the hypothalamic infundibulum in 1-, 8-, or 15-day-old chicks. Data are expressed as the mean ± SEM (n = 5−6). Asterisk indicates statistically significant difference versus vehicle (One-way ANOVA with Tukey's test as a post-hoc test: *P < 0.05, ***P < 0.005 vs. D1).

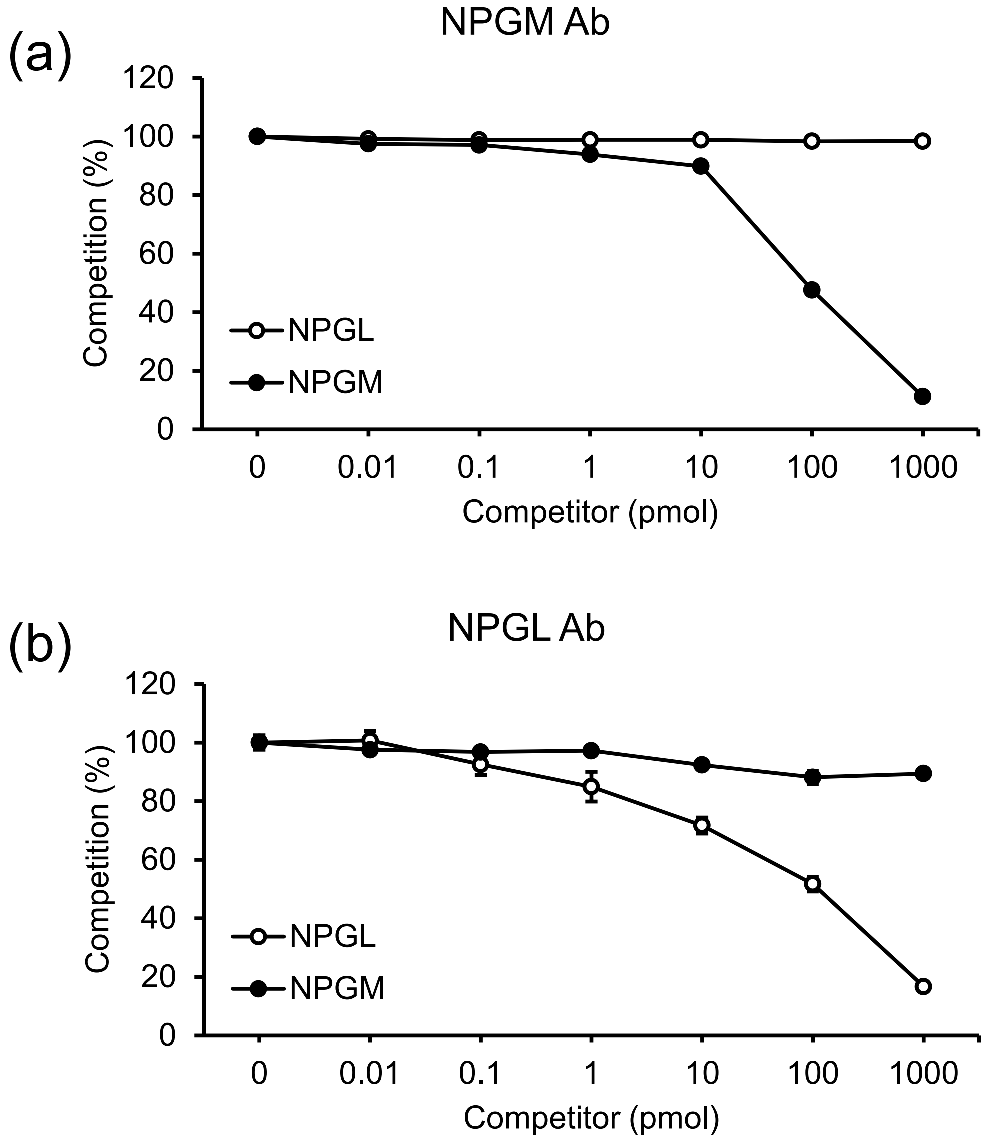

Supplementary Fig. S1

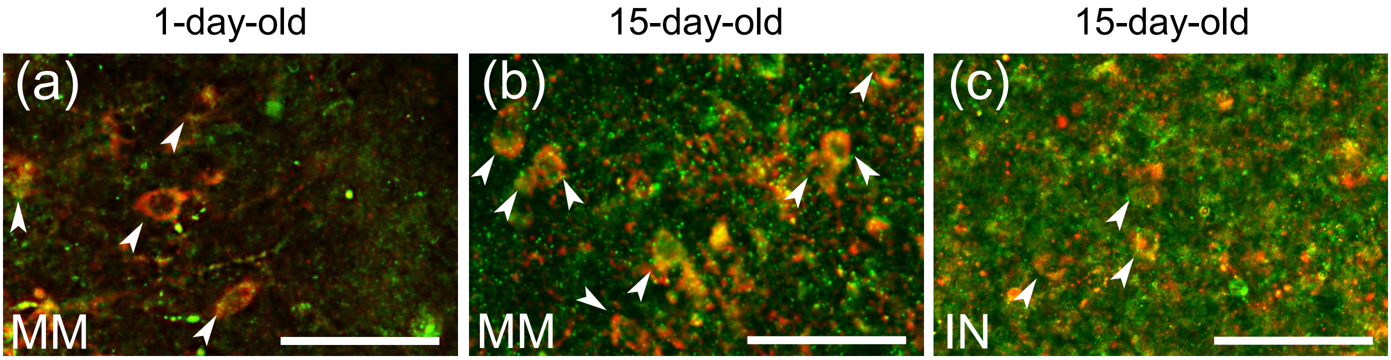

Supplementary Fig. S2

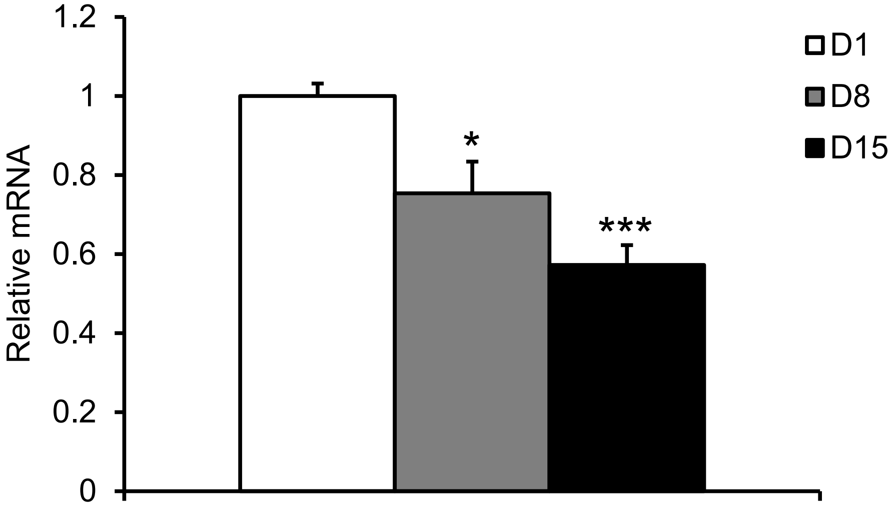

Supplementary Fig. S3

Supplemental Table 1-1. Sequences of oligonucleotide primers for real-time RT-PCR

gene	Forward primer	Reverse primer	Accession no.
NPGM	GTACGTAGACTGCGTGCTCT<br>(Nucleotide no. 285 to 304)	GTGAAAGCAGACCCTTGTAA<br>(Nucleotide no. 395 to 376)	XM_429770.2
NPGL	CTAGGAAAAAGACAGCTTGC<br>(Nucleotide no. 334 to 353)	CTTTCTTCGTCAGAACTGGT<br>(Nucleotide no. 454 to 435)	AB909129
HDC	GTAAGAAGTGATCCGCTGTT<br>(Nucleotide no. 1226 to 1245)	TGCTCAGTTCTTTCAGGAGT<br>(Nucleotide no. 1337 to 1318)	AB900795
ACTB	AGCCAACAGAGAGAAGATGA<br>(Nucleotide no. 339 to 358)	CCAGAGTCCATCACAATACC<br>(Nucleotide no. 467 to 448)	NM_205518.1

Supplemental Table 1-2. Sequences of oligonucleotide primers for *in situ* hybridization

gene	Forward primer	Reverse primer
NPGM	ATGGAATTCATGTGGAAGAG<br>(Nucleotide no. 1 to 20)	AGCATCTACAGTAAATGCTG<br>(Nucleotide no. 493 to 474)
NPGL	GGAGCGCCGAGTGAC<br>(Nucleotide no. −18 to −3)	CTTTCTTCGTCAGAACTGGT<br>(Nucleotide no. 472 to 453)
HDC	TGGAGATATGCTGGCTGATG<br>(Nucleotide no. 270 to 289)	ATGGGCACTAGGCCTTTCTT<br>(Nucleotide no. 713 to 694)

Supplementary Table 1
